# The influence of porosity and structural parameters on different kinds of gas hydrate dissociation

**DOI:** 10.1038/srep30324

**Published:** 2016-07-22

**Authors:** S. Y. Misyura

**Affiliations:** 1 Institute of Thermophysics Siberian Branch, Russian Academy of Sciences, 1. Akad. Lavrentyev Ave., Novosibirsk, 630090, Russia

## Abstract

Methane hydrate dissociation at negative temperatures was studied experimentally for different artificial and natural samples, differing by macro- and micro-structural parameters. Four characteristic dissociation types are discussed in the paper. The internal kinetics of artificial granule gas hydrates and clathrate hydrates in coal is dependent on the porosity, defectiveness and gas filtration rate. The density of pores distribution in the crust of formed ice decreases by the several orders of magnitude and this change significantly the rate of decay. Existing models for describing dissociation at negative temperatures do not take into account the structural parameters of samples. The dissociation is regulated by internal physical processes that must be considered in the simulation. Non-isothermal dissociation with constant external heat flux was simulated numerically. The dissociation is simulated with consideration of heat and mass transfer, kinetics of phase transformation and gas filtering through a porous medium of granules for the negative temperatures. It is shown that the gas hydrate dissociation in the presence of mainly microporous structures is fundamentally different from the disintegration of gas hydrates containing meso and macropores.

Gas hydrate deposits contain huge reserves of natural gas, and their intensive development will be started in the next decade^1^,^2^. Safety and environmental issues becomes one of the most challenging area in the world energy industry^3^,^4^. Now, not only the questions about the cost of conventional technologies of natural gas production, including shale gas, become urgent, but also the environmental problems play the primary role. The studies of gas hydrates are closely connected with the problem of global warming. The release of methane from the permafrost leads to the climate changes^5^. Alternative energy technologies can effectively solve the environmental problems^6^. Among such alternative technologies, there are production and storage of natural and artificial methane hydrates and burning of combustible gas hydrates. Many efforts are spent not only for improving the efficiency of technologies of gas hydrate production, but also the fight against hydrate plugs in the pipelines. These plugs grow rapidly, leading to a sharp increase in hydraulic resistance and stop of the oil and gas flows. In this regard, a large class of hydrate inhibitors is applied. A new class of hydrophobic amino acids kinetic inhibitors can effectively fight hydrate formation^7^. Pectin (kinetic hydrate inhibitor) also has the unique properties^8^. Much attention is paid to the problems, related to transportation and storage of raw materials in large containers^9^,^10^,^11^,^12^,^13^,^14^. Pelleted methane hydrate can be stored for a long time due to the phenomenon of self-preservation at the annealing temperatures and the lowest rate of methane hydrate dissociation is achieved at the temperatures of 265–267 K^15^. The effectiveness of the above technologies depends on the depth of understanding the dissociation mechanisms, boundaries of phase stability and metastable state. It is important to understand the fundamental difference between disintegration of gas hydrates at positive temperatures and dissociation at temperatures below the freezing point. At positive temperatures, gas diffusion occurs through the film or layer of water and it is described by the well-known dependencies. The kinetic equation satisfactorily describes the experiment and determined kinetic constants are used for a wide range of problems^16^,^17^,^18^. At dissociation in a porous media, Darcy’s law and Corey’s model for permeability^19^ are used in addition to the kinetic equations. Since gas hydrate collapse is accompanied by cooling, it is necessary to take into account heat exchange with the environment^20^,^21^. The rate of gas hydrate formation and decomposition is limited by thermal inertia of the medium and the rate of gas diffusion in liquid and solid. The influence of environmental heat transfer on the mechanism of gas hydrate decay is considered in refs 22, 23, 24, 25. Both kinetics and heat transfer should be taken into account^26^. The effect of heat transfer during crystal growth and decay is particularly important for highly nonequilibrium systems^27^,^28^.

The physical pattern of gas hydrate disintegration at negative temperatures is much more complex, and to date there are no accurate models, describing this dissociation. The problem is that in this case we are faced with a variety of structures and morphologies of crystal surface. In this case, it has no sense to choose the kinetic constants, describing the decay, since the value of the dissociation rate in the temperature range from 190 K to 273 K is changed by five orders, and it depends on the external pressure, type of hydrate, and ice morphiology^29^. The surfaces of hydrated rocks of marine hydrate deposits, mined in various places (Dongsha Area of the South China Sea, Pearl River Mouth Basin), have different morphology (laminated, nodular, vein, disseminated) with different grain sizes^30^. In literature, a lot of attention is paid to the study of ice structures in application to gas hydrate dissociation. Gas and ice are formed at decay of gas hydrates. There are three main structures of gas hydrates: cubic structure (*sI*); cubic structure (*sII*); and hexagonal structure (*sH*). Natural gas hydrates have usually structures *sI* and *sII*. When gas hydrates dissociate at temperatures below zero, there is the variety of ice structures and surface morphologies. Today, more than 15 crystalline phases have been discovered for ice; each of them has a characteristic metastable area^31^,^32^. The most common structure of ice in the range of low negative temperatures is hexagonal ice *Ih*. The crystal structure of cubic ice *Ic*, obtained at very low temperatures, is less stable and with increasing temperature, it turns to hexagonal ice. It is characteristic that for the external pressure of 1 bar and temperatures below 230 K, near the melting point ice has a branched pore structure. At the annealing temperatures, there is an abnormally low rate of gas hydrate decomposition (the phenomenon of self-preservation) and the ice surface is covered by a solid ice crust. At that, the surface ice structures have complex and differing morphology (disseminated, ramified, domed), which depends on the pressure, temperature and type of gas hydrate. Often in literature, abnormally low dissociation is associated with the morphology of the surface ice structures^33^. When dissociating at the annealing temperatures, a thin and very strong ice crust is formed on the granule surface. There is high internal pressure under the ice crust and it prevents further decay of gas hydrate. It turned out that the ultimate strength of this crust is many times higher than that of coarse-grained polycrystalline ice and its grain size is about 10 μm^34^. The structural characteristics of the surface envelope are investigated in refs 15 and 34, 35, 36, 37. The effect of key factors on the mechanism of self-preservation and the metastable state is considered in papers^15^,^29^,^36^,^37^,^38^. Self-preservation for hydrate dissociation in water + diesel oil dispersion systems is presented by ref. 39. The behavior of combined hydrates at the annealing temperature is considered in paper^40^. The effect of the thickness of gas hydrate layer and pellet diameter on the rate of decay is shown in refs 41 and 42. At gas hydrate combustion, not only the ice crust is formed on the surface of granules layer, but a water film; at that, the velocity of flame propagation depends on the velocity of the forced air flow^43^. Self-preservation at methane hydrate combustion leads to a multiple decrease in the fuel flow. As a result, the injection parameter and stoichiometric ratio, affecting the rate of chemical reaction at combustion, change^44^.

The porosity can be formed at both destruction and formation of gas hydrates. Kuhs *et al*. in detail investigated the formation of meso- and macro-pores of gas hydrates using scanning electron microscopy^45^,^46^. The porous structures were also detected and studied in a small growing hydrate layer at the liquid-liquid interface^47^. Natural gas hydrates, formed both from the continental and seafloor, are also indicate the presence of microporous structures^48^. The occurrence of porosity is associated with an excess of gas molecules at the time of gas hydrate formation^49^. Porous gas hydrates themselves in nature are formed in the pore space of a hard rock^50^,^51^. Coal moisture in porous leads to the formation of methane clathrate hydrates^52^. The formation of methane hydrates in coals increases the capacity of the coal to store gas. Methane storage capacity in gas hydrates is up to 2 orders of magnitude greater than the gas storage capacity of the coal by adsorption^53^. Formation and dissociation of gas hydrates in a porous medium are considered in refs 54 and 55. Mathematical models for reacting porous particles are widely used in problems of chemical engineering and combustion theory. Chemical transformations for porous particles in catalytic processes are considered in refs 56, 57, 58, 59. The effect of porous structures on chemical reactions at pyrolysis and solid fuels combustion are presented in refs 60, 61, 62, 63, 64, 65, 66, 67.

Currently, simulation (for negative temperatures) does not consider the diffusion and the filtration during dissociation and does not account structural parameters and as a result there is only a qualitative analysis. To model gas hydrate at negative temperatures, the following data are required: size distribution of granules; size distribution of pores, change in the ice crust thickness and pore density with time; heat flux value, as well as the values of pressure and temperature. For most experimental data obtained by now, only some of the mentioned parameters were measured. At that, the experiments are usually carried out under the quasi-isothermal conditions at a given temperature. The main purposes of the current study: to obtain the experimental data on methane hydrate dissociation in the presence of non-isothermal heat transfer and continuous change in the sample temperature (∆*Т* = const in the framework of one experiment and alteration of ∆*Т* in different experiments); to model dissociation using a new approach with consideration of heat transfer, dissociation kinetics and gas diffusion through the porous structures of samples; investigations of the influence of the pore size on the dissociation of methane hydrate granules and the decay of methane clathrate formed in the pores of coal; investigation of differences in the dissociation of gas hydrates in the presence of various pores (microporosity, meso and macroporosity).

## Experimental Data and Discussion

**Figure 1:**
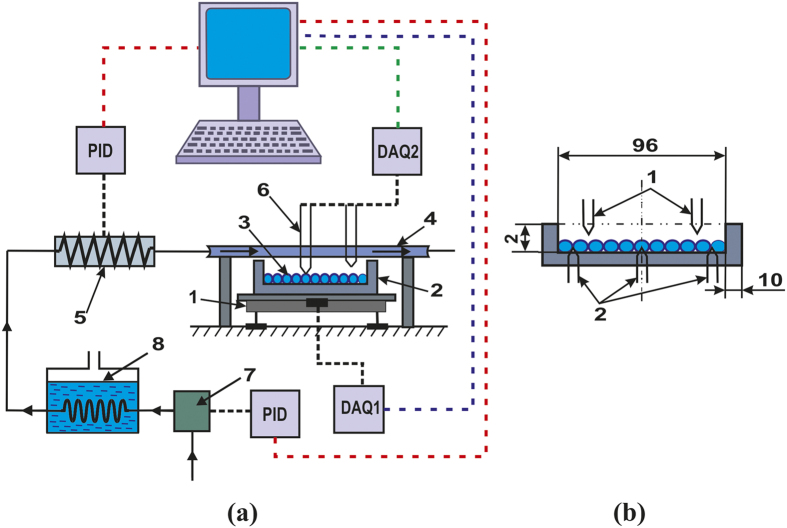
(**a**) Scheme of setup; (**b**) container with methane hydrate powder.

The scheme of setup and experimental section are shown in Fig. 1(a,b)).

When the pressure and temperature deviate from the equilibrium, dissociation of gas hydrate starts and methane escapes from the powder. A decrease in the sample mass is registered by digital scales 1. The powder of methane hydrate 3 in the form of one granules layer of a fixed size is located in container 2. Compressed air of the given temperature is fed via metal heat exchanger 4 from heater 5. Air from the compressor 7 is fed into a Dewar 8 for cooling. Thermocouples 6 measure the temperature of powder surface and gas above the sample. Thermocouples 1 (Fig. 1(b)) are between the heat exchanger and powder, and they measure the gas (methane) temperature *T*_*0*_. Before the experiment, above the working section there was air under the pressure of 1 bar. During the experiment, the pressure inside the working setup was kept constant (1 bar), using a pressure regulator. Thermocouples 2 (Fig. 1 (b)), measuring the powder temperature, were located in the sample layer on the axis and near the lateral walls and allowed determination of the average sample temperature. The total error of temperature measurements, taking into account the adjustment error, varied within 0.5–0.9 °C. Diameter of the cylindrical container 2 (a) was 96 mm, container height (b) *h* = 2 mm and the wall thickness of the tank (b) was 10 mm. The tank was manufactured from insulating material. The temperature difference between the gaseous environment above the powder and the sample (Δ*T*) was kept constant automatically. Methane hydrate granules were produced by the cooled autoclave. Fine crushed powder of ice was placed in this autoclave. Methane was fed into the autoclave and methane pressure 5–5.5 MPa was set (*T* = 0.5–1 °C). The granules, whose diameter varied in the range of *d* = 0.9–1.2 mm were used in experiments. During the experiment, the powder was presented by a single layer of granules. The reservoir with methane hydrate powder was stored in the Dewar vessel. The initial gas temperature above the reservoir was *T* = 220 K and pressure was 0.6 MPa. When the initial temperature was stabilized the reservoir pressure was reduced to 1 atm. At heating the powder emitted gas and the sample mass changed; this was registered by the digital balance. The total mass of remote gas corresponded to mass concentration of methane. Initial methane mass concentration was 12.41%. The structure of methane hydrate corresponded to sI with the elementary cell formula 2D·6T·46H_2_O (2(5^12^) + 6(5^12^6^2^)). The cell consists of 46 water molecules, six large and two small cavities. Porous characteristics are measured by the scanning electron microscopy. The average pore diameter was 0.8 μm. The size of ice grains was 10–50 μm. The density of open pores distribution before self-preservation was about 5·10^11^–7·10^11^ m^-2^ and 1·10^8^–1·10^9^ m^−2^ with partial self-preservation. The test coal had parameters: density (kg/m^3^) was 1490; coal moisture (wt %) was 29.5. Coal powder was produced by crushing and sieving (the average particles diameter was 1–1,5 mm). Powder X-ray Diffraction profiles show formation of methane hydrate (type sI) at 165 K. Low-pressure nitrogen and carbon dioxide adsorption used to determine pore size distribution. Large pores had the average diameter of about 50 nm.

**Figure 2:**
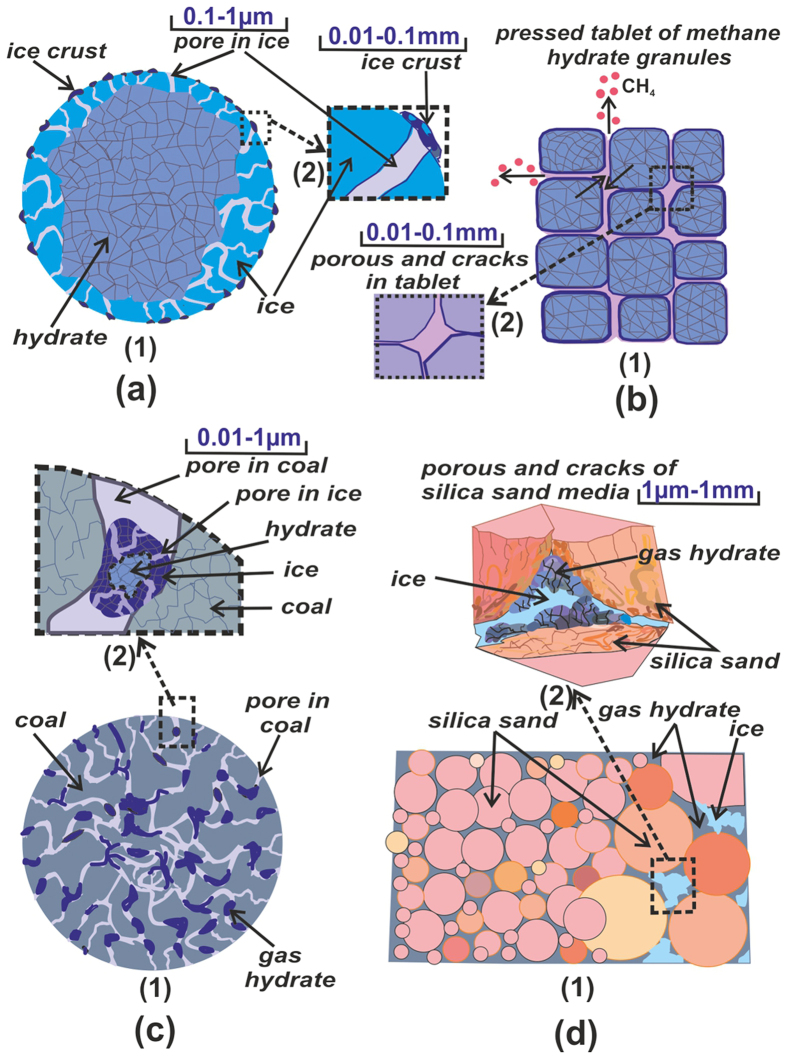
Scheme of gas hydrate dissociation at negative temperatures: (**a**) for granule; (**b**) for pressed tablet; (**c**) gas hydrate dissociation within coal pores; (**d**) dissociation within porous silica sand.

A wide range of problems in relation to gas hydrates is indicated in the Introduction. At sub-zero collapse temperatures, we have to deal with different types of gas clathrates and crystals with different morphologies. Depending on the characteristic scales of medium, porous matrix, hydrate particles and pores, different decomposition mechanisms can occur. Several specific types of gas hydrates with different physical scales, which should be considered at simulation, are shown in Fig. 2.

The sizes of natural and synthetic granules (Fig. 2(a)), used in process applications, usually vary from 50 μm to 5 mm. At the very beginning of decay, the granule surface is coated with open pores with the sizes from 0.1 to 1 μm. At the annealing temperatures, the pores surface and granules are covered with a thin ice shell with the thickness of 0.01–0.1 mm. With time, the thickness of the ice cover increases and the predominant part of pores becomes closed. Methane diffuses through the ice cover via the closed pores, and gas is removed through the open pores by filtration. The cellular structures (grains) with the size of about 10–50 μm are formed on the ice surface. Such microcellular ice cover has great strength, keeps the methane in the pores at high equilibrium pressure and inhibits rapid decomposition of gas hydrate. For large granules in the presence of external forced flow of gas and external heat transfer, it is also necessary to solve the equations of energy and thermal conductivity. Heat transfer should take into account the size of external environment, for instance, the thickness of the boundary gas layer. Pelleted gas hydrate (Fig. 2(b)) is obtained by pressing the individual granules. In this case, at dissociation filtration of two types should be modeled: inside the porous spaces of a granule and porosity of the tablet (pellet) itself. Both filtration rate and thermal conductivity inside the pellet will depend on porosity. The sizes of tablet pores are several orders larger than the granule porosity, but at a large height of the pellet, filtration resistance and thermal inertia should be also taken into account. In recent years, the increased attention is paid to gas hydrates, formed on the moisture inside the coal pores (Fig. 2(c)). Dimensions of coal pores vary in an extremely wide range of 1–1000 nm^53^. However, in very small pores of coal water drops cannot form due to the high capillary pressure. Therefore, not all pores contain gas hydrate. The amount of methane in the form of gas hydrate can be many times greater than the amount of gas adsorbed in the pores. This fact should be considered not only for the technological purposes, but also for safety reasons. It is necessary to calculate the dissociation rate to evaluate sudden methane emissions from mines, evaluate the risks of critical methane concentration in the air and possibility of its explosion. When modeling this type of dissociation, it is also necessary to consider the porosity of a hydrated particle, as well as coal porosity. A wide range of problems is associated with the hydrate porous medium in the following forms: porous medium of silica sand (d); huge amount of natural gas hydrates on the deep ocean floor and as permafrost. The heat transfer rate in such a matrix depends not only on the porosity of hydrate and entire matrix, but also on the contact area between hydrate and silica sand particles. Reduction in the area of permafrost on the Earth could significantly accelerate global warming due to the release of huge amounts of methane. Thus, kinetics of natural gas hydrate decay will vary greatly at ice formation in the porous matrix and in the event of self-preservation.

Dissociation at positive temperatures *T* > 0 °С occurs on the granule surface without pore formation and it depends on activation energy, pressure and temperature differences (system deviation from the equilibrium) and powder area^16^. The dissociation rate is quasi-constant for the most period of decay. Decay of gas hydrates at negative temperatures *T* < 0 °С is different, and its simulation is much more complex and currently there are no reliable calculation methods. At that, the temperature region is divided into three typical physical ranges. In this paper, we consider non-isothermal decay of methane hydrate, when the powder temperature changes from the temperature of liquid nitrogen to the melting point of ice under the external pressure of 1 bar. At that, temperature difference ∆*T* (∆*T* = *T*_0_ − *T*_s_, *T*_0_ is the ambient gas temperature, *T*_s_ is the powder temperature) is kept constant in each experiment. ∆*T* changes in different experiments. In this case for gas hydrate granules, we pass several heat regimes. We performed numerical simulation using filtration-kinetic model of dissociation. We used only one powder layer with the granule diameter *d* = 0.9–1.2 mm. Therefore, the powder behavior is determined as the total behavior of independent particles. Then, in the dimensionless form *m*/*m*_0_ (*m* is the initial mass of powder, *m*_0_ is the final mass of powder after gas hydrate decay) the behavior of a separate particle and powder layer will be the same. For the gas mass flow it is necessary to multiply the mass of a single particle to a value that the initial powder mass and the particles mass in the simulation will be equal. Dissociation occurs under the external atmospheric pressure of 1 bar. The calculations also used the following data. The average diameter of a particle (granule) is *d* = 1.05 mm. Initial methane concentration in a particle is 12.41%. The average granule pores diameter is 0.8 μm and for coal pores *d* = 50 nm, and the pore density changes from 6·10^11^ m^−2^ for the first regime (*T* < 228 K) to 1·10^8^ m^−2^ for the second annealing regime and then increases again up to 6·10^11^ (the third regime corresponds to creep, when 270 K < *T* < 273 K). The heat transfer coefficient *α*_*e*_ = 5.2 W/m^2^K was determined using the expression for the effective heat transfer coefficient for the layer (excluding radiation)^68^. Heat transfer between the gas medium and granule is determined by heat transfer coefficient *α*_*e*_ and temperature difference ∆*T*. The filtration mechanism inside the granule is described by Darcy relationship. The expression for heat balance of a separate particle (an ideal spherical granule) with the diameter of 1.05 mm will take form (1)


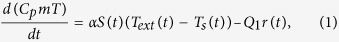


where *c*_*p*_ is average specific heat capacity (J/(kgK)) (an average value for ice and hydrate), *m* is the mass of particle (kg), *T* is average temperature of particle (K), *T*_*s*_ is particle surface temperature, *T*_0_ is ambient temperature (∆*T* = *T*_0_ − *T*_*s*_ = const), *t* is time (s), *S* is the area of granule surface (*m*^2^), *α*_*e*_ is heat transfer coefficient (W/(m^2^K)), *Q*_1_ (*Q*_1_ = 1020 kJ/kg)^69^,^70^ is heat of phase transformation at methane hydrate dissociation, *r* is the phase transformation rate of gas hydrate into methane and ice (kg/s). The equation for mass balance is written as (2)


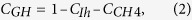


where *C*_*GH*_, *C*_*Ih*_, *C*_*CH4*_ are mass concentrations of methane hydrate, ice and methane. For the dissociation kinetics without filtration (no pores), we use (3)^16^,^17^,^18^


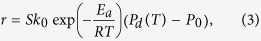


*P*_*d*_(*T*) is dissociation pressure of CH_4_ gas at temperature *T*; *P*_0_ is pressure of CH_4_ gas in the surrounding atmosphere, *S* is the surface area, *k*_0_ (*k*_0_ = 3.6·10^10^ mol/(m^2^·MPa·s))^16^ is pre-exponential factor, *E*_*a*_ (*E*_*a*_ = 78,4 kJ/mole)^16^ is activation energy at phase transformation. The equilibrium pressure depends on the temperature in the form of (4)^71^,^72^,^73^.


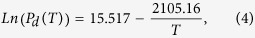


where *P*_*d*_(*T*), kPa, the temperature corresponds to range 148.8–262.3 К. For the higher temperatures, relationship (5) was used^69^.


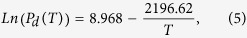


the temperature corresponds to range 260–273 К. Methane is filtered through the ice pores of the granule by the Darcy law (6)


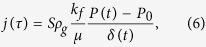


where *j* is the gas mass flow from the particle surface (kg/s), *S*(*t*) is the area of filtration (surface of granule, m^2^), *ρ*_*g*_(*T*) is density of gas (kg/m^3^), *P*(*t*) is pressure inside the pore (Pa), *δ* is the ice crust thickness (m), *k*_*f*_ is permeability coefficient, dynamic viscosity *μ* was determined by relationship in ref. 74. The permeability coefficient depends on pore diameter *d*_*p*_ and pore density *σ*_*p*_, according to ref. (7)^68^


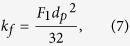


*F*_*1*_ is a part of the surface occupied by open pores, it is expressed by surface pore density *σ*_*p*_ (number of pores per 1 square meter (m^−2^)) by (8)


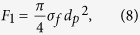


**Figure 3:**
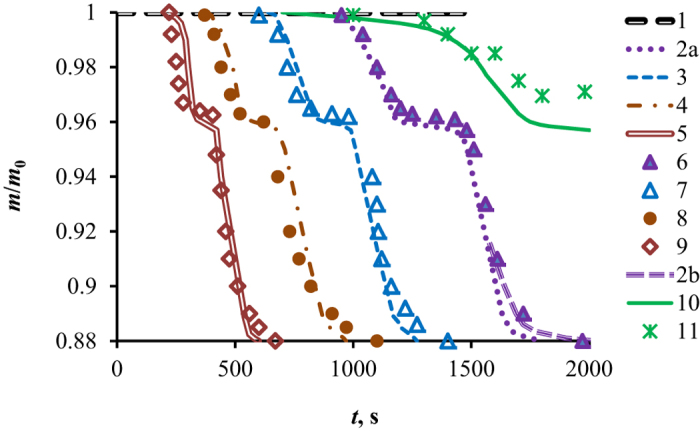
Dimensionless mass of methane hydrate (*m*_*i*_*/m*_*0*_) vs. time with self-preservation (1–9 for the artificial gas hydrate; 10–11 for the coal particles): simulation 1–5; 1 – ∆*T* = 5 °C; 2a–10 °C (1 self-preservation section); 2b–10 °C (2 self-preservation section); 3–15 °C; 4–25 °C; 5–40 °C; experiment 6–9: 6 – ∆*T* = 10 °C; 7–15 °C; 8–25 °C; 9–40 °C; 10 – simulation for coal (∆*T* = 15 °C); 11–experiment for coal (∆*T* = 15 °C).

The simplified scheme of dissociation is used for simulation, when ice crust thickness *δ* is distributed uniformly over the circumference of a cylindrical granule. Simulation data on *m*_*i*_/*m*_0_ with self-preservation for the second region and without self-preservation for the first and third regions are shown in Fig. 3. There are four specific time intervals from the experiment: (1) high rate of decay due to high pore density, (2) the region of self-preservation; (3) high rates of decay and less noticeable effect of self-preservation, (4) a significant decrease in the rate of decay at the end of decomposition.

At ∆*T* = 10 °C, simulation was performed for one region of self-preservation (curve 2a). Simulation for two regions of self-preservation is presented by curve 2b. Experimental data correspond better to curve 2b, and this proves a significant decrease in the pore density at the end of the third region of dissociation (excluding the zone of ice creep near the melting point). A noticeable deviation of experimental data from calculation is observed near *m*/*m*_0_ = 1. The calculation curve has the value of derivative for curve *dm*/*dt* is much smaller than the experimental curve. Perhaps, this is due to a higher rate of gas filtration through the pores, when the thickness of the ice crust tends to 0. It is obvious that for this case, the Darcy equation can be non-linear. A significant deviation of experiment from simulation for coal (curve 10) is also quite logical. To date, there are no reliable data on how water or hydrates are distributed in the pores of different sizes. It is necessary to know gas hydrate distribution over the volume of coal particle. However, it can be clearly seen that the dissociation rate for coal particles is significantly lower than for the granules consisting only of gas hydrate. The lower dissociation rate is certainly connected with a small size of pores in coal and with high thickness of the ice within the coal pore. A similar result can be obtained at dissociation of a porous hydrated matrix (Fig. 2d) at *T* < 0 °C and with self-preservation. A particularly low rate of decay will be observed for not broken unified rock with great height and small size of cracks in the rock. To simulate this process, it is necessary to know not only the porosity and rock composition, but also size distribution of pores and cracks.

**Figure 4:**
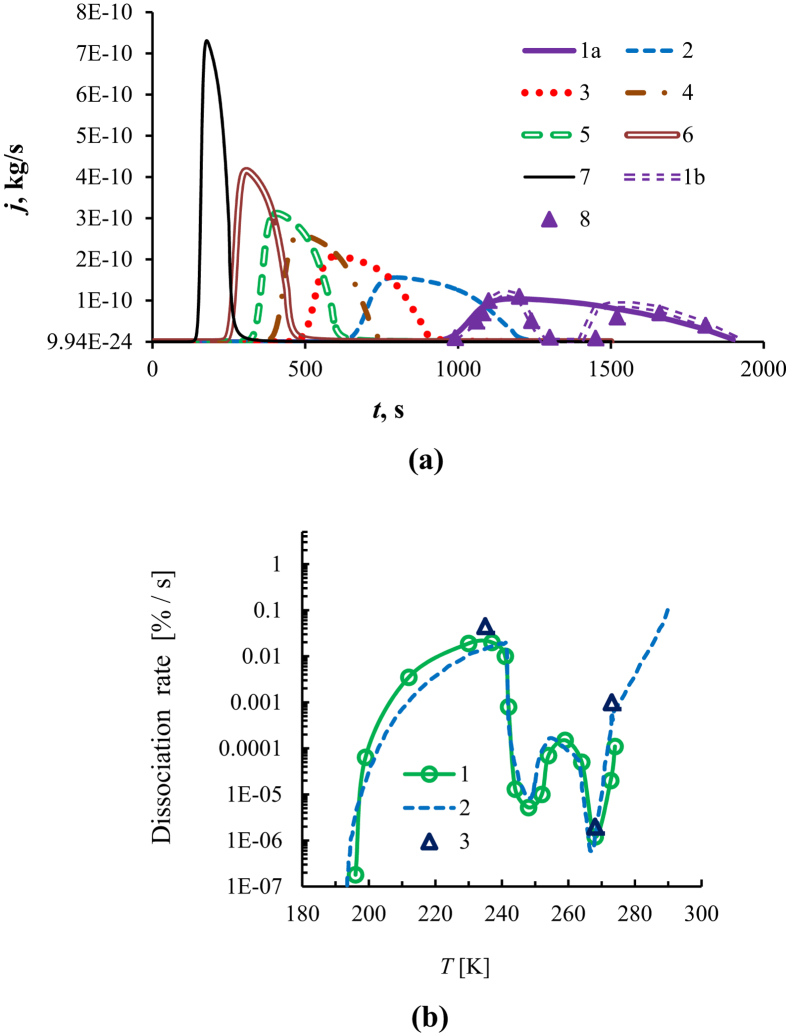
(**a**) Methane mass flux vs. time: simulation 1–7: 1a – ∆*T* = 10 °C (without self-preservation); 1b – ∆*T* = 10 °C (with self-preservation); 2–15 °C; 3–20 °C; 4–25 °C; 5–30 °C; 6–40 °C; 7–70 °C; experiment 8 – ∆*T* = 10 °C. (**b**) Dissociation of methane hydrate: Curve 1(experiment from presented paper); Curve 2 (experiment from refs 15 and 77); 3 – simulation.

The calculated curves and experimental points for dissociation rate *J* are shown in Fig. 4a. It is evident that *J* has strongly non-linear character vs. time: it increases first, reaches the maximum and then decreases. At the final stage of dissociation, the derivative *dJ*/*dt* tends to zero, and this is associated with the spherical shape of a granule. When approaching the dissociation front to the center, the mass of emitted methane tends to 0^22^. This non-linear character of dissociation rate was obtained for the first time by simulation and it is in good agreement with the experimental data with consideration of self-preservation (points 8). The similar non-linear character vs. time and temperature was observed in experiments on gas hydrate combustion^75^,^76^.

According to simulation, dissociation rate depends on the pellet diameter, porosity and self-preservation temperature. In the annealing zone, the pore density depends strongly on the sample temperature and changes by several orders of magnitude, which leads to a change in the dissociation rate by 4–5 orders. Figure 4b shows the overall methane hydrate dissociation curves at the atmospheric pressure of 1 bar. Each point of the curve corresponds to a given constant temperature and it was obtained at loss of 50% of methane (i.e., the rate of 50% of methane loss per a certain time). Curve 1 corresponds to the experimental data of this study. Curve 2 corresponds to the experiment of refs 15 and 77.

Experimental points for (1) are obtained as an average value in several repeated experiments. The difference of the average dissociation rate (%/s) for each constant temperature (*T*_*i*_) on the average value (for the same fixed *T*_*i*_) is within (8–15%). At that, an error in determination of the dissociation rate is within 3–6%. The difference between the repeated experiments is determined by the fact that it is difficult to maintain the uniform size distribution of granules and that the geometric parameters of porosity vary. Even a slight change in the granule diameter (about 10%) affects significantly the dissociation rate because it is related nonlinearly to the initial diameter of a particle^78^. Some difference between the experimental values and simulation results can be caused by a slight change in the sample temperature at dissociation and inaccurate data on the value of pore density.

## Conclusion

To simulate the dissociation of gas hydrates, it is necessary to take into account heat transfer for both the quasi-isothermal case (while decaying the sample is cooled and heat supply is required) and non-isothermal conditions. For this, it is important to know the temperature difference between the sample and external environment and the heat transfer coefficient. Non-isothermal dissociation in a powder layer of gas hydrate at negative temperatures has four characteristic time regimes: (1) high rate of decay due to high pore density; (2) the region of self-preservation at the annealing temperature and the low rate of decomposition; (3) high rates of decay due to the growth of pore density; (4) a significant decrease in the rate of decay because of an increasing role of self-preservation. Dissociation at low temperatures is fundamentally different from dissociation at temperatures above zero due to formation of porosity and filtering mechanism. The dissociation processes differ significantly at a change in morphology of the surface structures and characteristic sizes of a porous medium. In this regard, kinetics of dissociation of natural gas hydrate for one granule, compressed pellet, coal and various sedimentary rocks, and porous media will differ significantly. Low coal pore diameter (10–100 nm) leads to the significant decrease in the rate of dissociation and in release rate of methane from the sample. The real natural and technical processes are nonstationary and non-uniform, and this should be taken into account at simulation of gas hydrate dissociation. Gas hydrate kinetics dissociation is determined not only by the dissociation front motion, but also by the pore density and their size distribution over the whole volume of the granules. When modeling non-isothermal dissociation for the negative temperatures, it is necessary to take into account heat transfer, kinetics of phase transformation and gas filtering through a porous media. More accurate simulation of the dissociation kinetics is required to improve the technology of long-term storage and transportation of gas hydrate. It will allow us to reduce the cost of technology. Storage efficiency is increased while maintaining the strength of the ice crust at a temperature maximally close to the ice melting point. The presented model assumes quasi-stationary thermal conditions, i.e., a slow change in the powder temperature versus time. At high rates of heating or cooling and a thick layer of powder or porous rock, it is necessary to solve the heat conductivity equation for the solid and gas phases, and this is the subject of further research. To improve simulation accuracy, additional experimental studies are required to determine pore distribution and derive experimental dependence of pore density on temperature at self-preservation, as well as when approaching the melting point. This modeling approach is important for solving the problems of storage and transport of gas hydrates and improving technological efficiency. It is also important for the assessment of the risk of explosion at transportation of raw materials in tankers and sudden release of methane in coal mines as well as for the development of the global warming models.

## Additional Information

**How to cite this article**: Misyura, S. Y. The influence of porosity and structural parameters on different kinds of gas hydrate dissociation. *Sci. Rep.* **6**, 30324; doi: 10.1038/srep30324 (2016).
